# How to Include Patients' Perspectives in the Study of the Mind: A Review of Studies on Depression

**DOI:** 10.3389/fpsyg.2021.651423

**Published:** 2021-04-12

**Authors:** Henriette Löffler-Stastka, Kathrin Bednar, Ingrid Pleschberger, Tamara Prevendar, Giada Pietrabissa

**Affiliations:** ^1^Department of Psychoanalysis and Psychotherapy, Medical University of Vienna, Vienna, Austria; ^2^Vienna University of Economics and Business, Vienna, Austria; ^3^University of Applied Sciences BFI Vienna, Vienna, Austria; ^4^Sigmund Freud University Vienna - Ljubljana Branch, Ljubljana, Slovenia; ^5^Department of Psychology, Catholic University of Milan, Milan, Italy; ^6^Istituto Auxologico Italiano IRCCS, Psychology Research Laboratory, Milan, Italy

**Keywords:** mental disorders, depression, phenomenology, first-person perspective, lived experience (of the illness), clinical psychology

## Abstract

Depression has been widely studied by researchers from different fields, but its causes, and mechanism of action are still not clear. A difficulty emerges from the shifting from objective diagnosis or analysis to exploration of subjective feelings and experiences that influence the individuals' expression, communication and coping in facing depression. The integration of the experiential dimension of the first-person in studies on depression–and related methodological recommendations–are needed to improve the validity and generalizability of research findings. It will allow the development of timely and effective actions of care. Starting from providing a summary of the literature on theoretical assumptions and considerations for the study of the mind, with particular attention to the experiential dimension of patients with depression (aim #1 and #2), this contribution is aimed to provide practical suggestions for the design of research able to incorporate first- and third-person accounts (aim #3). It is also aimed to review qualified phenomenological methods for the acquisition and interpretation of experiential data in patients with depression (aim #4). Recognizing the first-person perspective in the study of depression is a major step toward a better understanding and treatment of this disorder. Theoretical constructs and technique suggestions that result from this review offer a valid starting point for the inclusion of the experiential dimension to common third-person research in the study of the mind.

## Introduction

There is an ongoing discussion in psychiatry on the definition of depression and its subtypes. Many factors can cause depression and are tied to other elements of one's own health (Wakefield and Schmitz, 2013; Ratcliffe, 2015). Healthcare providers are often unable to determine what is causing depression, and this prevents them from structuring adequate psychological interventions alone or in conjunction with antidepressants (Holsboer, 2010).

Recently, the complex nature of depression and the need to understand its features on multiple levels has been emphasized by researchers from different fields, including neurobiology (Barch, 2013), psychology (Scott, 2009), psychiatry (Parnas and Zahavi, 2002; Biancosino et al., 2010; Wakefield and Schmitz, 2013; Fuchs, 2014), health sciences (Telford et al., 2011; Coventry et al., 2014) and phenomenology (Ratcliffe, 2015).

*Phenomenology* points at the importance of the first-person account and offers a new method to access experience. Stemming from philosophical tradition, phenomenology provides a paradigm useful for the development of valid methods to target subjective and interpretative experiences–which is also a central aim for favorable outcomes in psychotherapy.

In 1996, Varela proposed the term *neurophenomenology* to designate a new approach to the study of the mind that adds phenomenological methods to the traditional third-person account (electrophysiological measures, neuro-imaging techniques, etc.)–thus promoting a systematic way to explore the subjective experience (Varela, 1996). The *neuro* prefix is not limited to neuroscientific methods but refers to cognitive sciences in a broader sense. Varela (1996) idea is to establish a new systematic and disciplined methodology that links mental correlates to experience. In this line, other authors claim that the phenomenological approach allows a better understanding of depression, which in turn leads to more successful diagnoses and treatments (Parnas and Zahavi, 2002; Granek, 2006; Rhodes and Smith, 2010; Gallagher and Zahavi, 2012; Sandhu et al., 2013; Coventry et al., 2014; Fuchs, 2014; Ratcliffe, 2015).

den Boer et al. (2008) discuss the usage of mixed-methods approaches, as e.g., neurophenomenological studies use neuroscientific techniques (fMRI, EEG, PET, SPECT) in conjunction with first-person methods. They conclude that not only the combination of first-person and third-person data is feasible, but that inclusion of the first-person perspective favorably influences the outcome of neuroscientific experiments profoundly (Ciechanowski, 2015). Dzhambov (2015) provided further evidence for the importance to include the qualitative dimension in third-person studies. The author argues that psychopathological phenomena are not clearly defined and should be more deeply explored directly with the person by means of qualitative methods.

Efforts to integrate the classical phenomenological criteria of validity with the standards of empirical research were made. Classical assumptions of phenomenological psychopathology have been operationalized in clinical descriptive manner (e.g., Tellenbach, 1980; Berner et al., 1983) and used to develop standardized assessment tools used in empirical research (e.g., DSM system) (compare Doerr-Zegers et al., 2017).

To enrich clinical decision making (Wadowski et al., 2015, 2019; Seitz et al., 2017) by bridging the gap between neuroscientific methods and first-person perspective (Löffler-Stastka and Parth, 2013; Parth et al., 2014) via psychotherapy research (Stanghellini, 2019), there is a need for more clarity on assumptions and methods of neuro/phenomenological approaches to the study on the mind.

To address this issue, this study aims for the first time to provide a summary of the literature on theoretical considerations for the study of the mind with particular attention to the experiential dimension of patients with depression (aim #1 and #2).

Aim #1 targets at arguments for the inclusion of the experiential dimension in the study of the mind and depression, while
aim #2 zeros in on the criticism against the inclusion of the experiential dimension in the study of the mind. The present contribution also
aim #3s to provide practical recommendations for research-designs able to incorporate first^1^- and third-person accounts, and
aim #4, to review methods for the acquisition and interpretation of experiential data in patients with depression in order to–all in all–strengthen public/patient involvement.

## Method

### Definition of Terms

This article uses the term (subjective) experience in accordance with the definition by Parnas and Zahavi (2002, p. 145), who describe experiences as conscious states that are combined with subjective feelings and meanings. This subjective side is also stressed by Varela and Shear (1999, p. 1). We use the terms *experience, subjective experience, first-person experience, direct experience, first-person account*, and *first-person perspective* synonymously. As stated by Varela (1996, p. 331) the terms *third-person perspective, third-person account*, etc. refer to the study of natural phenomena or science of mind. Varela and Shear (1999) stress that third-person data are never entirely objective as the subjective is already implicit in the objective according to a social-constructivist view. Therefore, we avoid terminology that focuses on the split between subjective and objective in this review. Although the terms first-person and third-person data fall under the same misconception, we use them as a helpful and common distinction.

### Search Strategy

In order to find and examine studies that target phenomenological component, literature search was performed using a deductive-inductive approach (e.g., the results of the initial search guided following search for papers and decisions on their inclusion in the analysis and qualitative summary. The search for the papers was conducted in several databases: PubMed, Taylor & Francis Online, Wiley Online Library, SAGE journals, ScienceDirect, BioMed Central, and JSTOR databases from 01/01/1945 to 01/02/2020 during April-July 2020.

The search strategies combined key terms for the concepts of “*depression/ major depressive disorder-MDD*,” “*phenomenology/neurophenomenology*,” “*experience, subjective experience, first-person experience, direct experience, first-person account, first-person perspective*,” and “*third-person perspective, third-person account*” using thesauri and Subject Headings (for PubMed). Boolean and truncation operators were used to more systematically combine search terms and to list documents containing variations on search terms, respectively (Johnson, 2002). The search syntax was modified as appropriate for each database (see Supplementary Table 1 for detailed information on the search strategy).

Literature search was conducted targeting articles that (1) discuss arguments for the inclusion of the experiential dimension in the study of the mind and depression or (2) present criticism against this endeavor. Additionally, we included articles that (3) show how first- and third-person accounts can be combined by presenting important aspects concerning the general study design, the choice of participants, as well as the required skills and qualifications of the researchers. Finally, (4) we focused on methods that can be used to acquire and analyze experiential data.

First, articles discussing the practical use of- and theoretical arguments for the use of neurophenomenological method in different contexts were searched (key terms: “neurophenomenology” and “method”). Second, the search focused on articles discussing the need for the inclusion of phenomenological approaches in the study on depression. Since the key terms “neurophenomenology” and “depression” yielded a very few results, the term “phenomenology” was used instead. The search was limited to relevant articles including depression and phenomenology in their abstract. Further literature was added by including relevant referenced articles and manual search (“snowballing”). Then, the reference lists of all selected articles and relevant systematic reviews were manually screened to identify any additional reference for possible inclusion.

### Inclusion and Exclusion Criteria

Only original articles that reported studies that: (1) discuss arguments for the inclusion of the experiential dimension in the study of the mind and depression; (2) present criticism against this endeavor; (3) show how first- and third-person accounts can be combined by presenting important aspects of the (a) study design, (b) choice of participants, and (c) researchers' required skills and qualifications were included. Moreover, records (4) focused on methods that can be used to (a) acquire and/or (b) analyze experiential data were incorporated. No limitations were set for study design, language, ethnicity and the year of publication.

Notably, articles on depression were selected if focused on the need and use of experiential dimensions for the study, diagnosis, classification or treatment of this syndrome. Also, contributions were included if the presented method was thoroughly described and focused on the acquisition of experiential data, their analysis or on how the method itself can be included in a neurophenomenological framework combining first- and third-person data.

### Study Selection

Searches were as broad and as inclusive as possible. First, databases were searched with broad search words “phenomenology” and “depression/MDD/depressive disorder” as described above. After the first number of papers that searches yielded throughout the databases, snowball method was employed, and references of papers checked to further manually search for other relevant papers. Following the search and exclusion of duplicates and systematic reviews/metaanalyses, two reviewers (K.B. and I.P.) independently screened the eligibility of the articles first on the title and the abstract, and on the full text according to the inclusion criteria. Disagreements were resolved by discussion in the group with reviewer H.L.-S.

In similarity to Smith et al. (2011), the review team included at least one person with methodological expertise in conducting reviews (G.P., T.P., and K.B.) and at least two experts on the topic under review (H. L.-S. and G.P., T.P.).

The number of articles that were originally selected as relevant was reduced from 151 articles to 59 final articles in the inductive, second phase. Here, two of the authors (K.B. and I.P.) selected articles that focused on the neurophenomenological method by either presenting a pilot study, discussing the constraints of other methods or focusing on the theoretical benefits of the method. This was done through an initial titles and abstracts–screening of the recalled articles within literature research on neurophenomenology as a method. Articles about depression were selected, if they focused on the need and use of the inclusion of an experiential dimension in the study, diagnosis, classification or treatment of depression. Articles presenting different methodological approaches were included if the approach was thoroughly described and the method focused on the acquisition of experiential data, its analysis or on how to include the approach in a neurophenomenological framework combining first- and third-person data. Both raters explored full texts of the eligible papers and shared decisions concerning article inclusion were made.

## Results

The following headings represent summarized literature, arguments and frameworks discussed in the reviewed articles. Starting from enclosed theoretical considerations (aim #1 and #2), practical recommendations for the design of a research study that incorporates first- and third-person accounts (aim #3), as well as methods that are qualified for the acquisition and analysis of experiential data (aim #4) are then presented.

### Aim #1: Arguments for the Inclusion of the Experiential Dimension in the Study of the Mind

Research emphasizes that it is important to include experience in the study of the mind, and warns that reductionist approaches interested only in behavioral measure (Hartelius, 2007, p. 24) strongly affecting the *fields* of psychiatry (Sass et al., 2011), psychology (Hartelius, 2007; Weger and Wagemann, 2015) and psychoanalysis (Cusumano and Raz, 2014; Yovell et al., 2015) as this reductionism reflects only a one-sided view on reality (Weger and Wagemann, 2015).

The exclusion of the experiential dimension is further associated with general criticism against the modular, reductionistic and materialistic epistemology typical of neurosciences, and the biologization of subjectivity (Yovell et al., 2015, p. 4). Hartelius (2007) extends this criticism to empirical sciences that do build a better understanding of first-person perspective (p. 25).

Researchers argue that the purely neurocentric, cognitivist, and computationalist approaches that focus on brain and behavior should extend to the level of subjectivity and experience (Sass et al., 2011, p. 3), and employ a practical, rigorous and effective first-person methodology (Hartelius, 2007, p. 24) to gain a better insight on *psychopathological* phenomena (Hartelius, 2007).

Sass et al. (2011) claim that phenomenology can contribute to the study of psychopathologies, such as schizophrenia, by adding subjectivity that is not merely descriptive but explanatory. Other researchers (Akiskal et al., 2001, 2006) confirm the feasibility of phenomenological methods in psychiatric diseases such as bipolar disorder. Akiskal et al. (2001) examine in which way patient-oriented outcome research can implement conventional diagnostic procedures in case of mania.

Conventional diagnostic manuals and common guidelines for mental state examinations are based on the results of (clinical) neuroscience (Jablensky and Kendell, 2002; Holsboer, 2010) focused on genetics and biomarkers (Holsboer, 2010), and do not address patients' subjectivity or intersubjectivity (p. 140). Phenomenology would fill this gap and thus help to differentiate psychological traits from emotional states in psychopathology (Parnas and Zahavi, 2002, p. 158) and to improve psychiatric diagnosis and classification (Parnas and Zahavi, 2002, p. 159). A phenomenological approach could be relevant for redefining criteria for major MDD and normal-range distress or sadness. Prevalence estimates of DSM-defined MDD shows that threshold criteria in DSM for MDD are too low (Wakefield and Schmitz, 2013, p. 44). Further investigations phenomena associated with unipolar depression could also profit from the integration of first-person account, e.g., apathy and dysphoria (Biancosino et al., 2010).

Moreover, Varela and Shear (1999, p. 4) emphasize that experience does not only yield additional information or a better explanation of a phenomenon, but also provides suggestions to optimize *treatment outcome* and adds knowledge to moderator and mediator research on the processes of changes including psychotherapy. From a clinical point of view, e.g., treatment motivation is a known predictor for therapy outcome (Pihet et al., 2013). Subjectively experienced degree of distress as well as e.g., anhedonia is described as moderator for social functioning targeted in psychotherapy treatment (Allott et al., 2011).

Holsboer (2010) argues that the future of the treatment of depression lies in personalized therapy. This would require gene tests and biomarkers able to detect pathologic mechanisms prior to clinical symptoms manifestation. Early interventions would allow the *prevention* or slowing down of the disease onset. Depression severely affects experience (Ratcliffe, 2015) and is–in turn–severely affected by subjective experience–increased knowledge on phenomenological methods for early detection of depressive symptom would further increase treatment outcomes.

### Aim #2: Criticism on the Inclusion of the Experiential Dimension in the Study of the Mind

It is widely accepted by phenomenologists that reflection alters human experience. This argument is often taken as criticism against first-person methods (e.g., Weger and Wagemann, 2015). Still, Husserl (1982) (as cited in Sass et al., 2011, p. 11) argues that we do not have knowledge of lived experience before reflection, and that reflection should therefore be “imbued with a self-critical awareness of precisely such dangers.” Varela (1996) proposes phenomenological reduction as sophisticated and systematic way of exploring the structure of experience, and names four aspects describing the phenomenological reduction: attitude, intuition, invariants, and training. The attitude involves beliefs to allow investigation of reflection (Varela, 1996, p. 336-337). The attitude of reduction is the necessary stating point, similar to doubt sudden, transient suspension of beliefs (Varela, 1996, p. 336) should be reflected. The act of reflection, also in further developments of neurophenomenology, has to be investigated as an enactment of a lived experience toward and structured along radical neurophenomenology (Petitmengin et al., 2019). Through intuition a certain intimacy with experience is reached, allowing for intersubjectivity of descriptions of experience (which Varela calls “invariants”). Finally, Varela stresses that neurophenomenological knowledge can be achieved only if both researchers and study participants are properly trained to deepen attention, intuition, and amplification of the experience (Varela, 1996, p. 338).

Desbordes and Negi (2013, p. 1) outline that first-person data assessment methods are unreliable as psychic functions are–to a great extent–consciously unapproachable. But Petitmengin et al. (2013) argue that elaborated methods targeting the subjective experience–e.g., elicitation interviews (see below)–allow retrospective access to detailed aspects of the subjective experience.

Criticism does not only extend to phenomenological approaches and the reliability of first-person data, but also targets third-person approaches. In the field of psychoanalysis, some authors claim that neurosciences are essentially irrelevant (e.g., Blass and Carmeli, 2007; Yovell et al., 2015, p.9), as subjectivity is denied by cognitive scientists and neuroscientists in their research aims, attitudes and beliefs (Yovell et al., 2015, p.5). Not surprisingly, there is also no unified stance of psychoanalysts toward the field of neuropsychoanalysis, which tries to bridge the gap between psychoanalysis and neuroscience (Yovell et al., 2015). Yovell et al. (2015, p. 12) argue for collaboration between the two disciplines: neuroscience can inform psychoanalysis by complementing its knowledge about neural basis of specific disorders and symptoms including depression, while psychoanalysis can add to neurosciences the exploration of unconscious personal meanings (Yovell et al., 2015, p. 29).

### Aim #3: (Neuro)Phenomenological Research: Research- and Study-Designs

In the attempt to address the criticism Hartelius (2007, p. 28) makes to neurophenomenology to lack of useful methods, the following sections outlines approaches that try to overcome this problem.

#### The Study Designs

Gallagher (2003) reviews three phenomenological approaches to assess possible contributions of phenomenology to experimental cognitive neurosciences: neurophenomenology, indirect phenomenology, and “front-loaded” phenomenology. These approaches represent different concepts of phenomenology and propose distinct roles for it in the context of empirical science. Gallagher recommends to consider first-person experience in experimental settings either by training participants to report their experience reliably (neurophenomenology) or by taking insights obtained from previous (neuro-)phenomenological experiments into account in the setup and interpretation of experimental outcomes (front-loaded neurophenomenology). A concept close to Gallagher's front-loaded phenomenology is Weger and Wagemann (2015) approach aimed to complement experimental (psychological) research with first-person experience. Indirect phenomenology or–as named by Dennett (1991) (as cited by Gallagher, 2003, p. 90)–“heterophenomenology” represents a less formal version of phenomenology. Gallagher proposes to avoid this phenomenological approach because first-person data are not based on phenomenological analysis, but “averaged” or “washed out” (Gallagher, 2003, p. 90). Sass et al. (2011, p. 6) summarize the neurophenomenological approach (Varela, 1996; Lutz et al., 2002; Lutz and Thompson, 2003) as follow: subjects included in empirical test formats should be investigated via qualitative methods and open questions to reduce theoretical pre-assumptions. Further, these descriptions of experiences should be categorized, validated by different researchers, and then interpreted together with neuroscientific measurements, as also suggested by Berkovich-Ohana et al. (2020). Sass et al. (2011) also refers to Varela's concept of “reciprocal constraints” (Varela, 1996) to describe the interplay of phenomenology and neuroscience: phenomenology constrains neuroscience by providing hypotheses that can be used in neuroscientific studies, but the constraint is reciprocal in testing phenomenological findings empirically (Sass et al., 2011, p. 5). An example of neuroscience constraining phenomenology is a neuroimaging study revealing two mechanisms instead of a simple mechanism that was previously proposed; this would provide the opportunity for a phenomenological analysis of the phenomenon which could reveal true (Sass et al., 2011).

#### The Sample

The choice of the participants depends on the method used for acquiring experiential data. As described and proposed by Varela (1996), in neurophenomenological studies it is necessary to train participants in order for them to “gain greater intimacy with their own experiences” (Sass et al., 2011, p. 6). Desbordes and Negi (2013, p. 1) argue that research participants should have a background in contemplative practice (e g., meditation, or other introspective methods) that allow moment-by-moment description of experience (p. 1). Further, other researchers propose to include participants who have undergone psychoanalysis, as they assume them to be better rained in describing their subjective experience (Cusumano and Raz, 2014).

A different approach implies to train the interviewer to assist in the process of retrieving and describing experiences by leaving the objective third-person observer position and assisting opening the participants to their own individual experiences (Bockelman et al., 2013, p. 8). Moreover, Petitmengin (2006) presents an interview technique that enables the researcher to elicit subjective experience from untrained participants (see below).

The choice of participants can vary from the researchers themselves to experts, general population, or specific groups of individuals, depending on what experience most promisingly fits the purpose of the study (Wertz, 2005). The number of participants depends on the nature of the research problem. Although case studies hold the same validity of studies involving large samples (Wertz, 2005), it must be highlighted that detailed descriptions of subjective experiences retrieved from case reports, can hint more to the pathoplastic ingredients of a disorder than studies with simplified methods used to include a great number of participants (Parnas and Zahavi, 2002, p. 156).

#### The Researcher(s)

Bockelman et al. (2013) present “lessons learned” from previous neurophenomenological studies. First, all members of a team should possess a common lexicon and conceptual framework to be able to work together and synthesize results in an efficient way–as opposed to separate individual interpretations (Bockelman et al., 2013, p. 6). Therefore, researchers should be trained in neurophenomenological theories and methods, and regular meetings should be held for brainstorming and teaching to make sure that all team members have a full picture of the research design and goals. Neurophenomenological method should also help strengthening the doctor-client or interviewer- interviewed interaction. McInerney and Walker (2002, p. 183–184) emphasize that phenomenology can be used to complement standard neuropsychological assessment methods aimed at gathering symptoms–rather than their subjective meanings–within an inequal relationship between client and investigator that do not open for clients' insights. As a start for a collaborative relationship with the client, the researcher must establish a dialogue by asking the person to give examples for situations where s/he has experienced a certain difficulty. Accordingly, McInerney and Walker (2002, p. 184) argue that clients should be encouraged to actively contribute to their own understanding of a specific situation. Moreover, Desbordes and Negi (2013) stress that neurophenomenology benefits from participants but also from researchers that are trained in contemplative practice. Similarly, Weger and Wagemann (2015, p. 40) suggest that investigators should use the introspective method as it does not only offer ideas and insights for new exploration, but also helps professionals to develop a critical view on decisions (e.g., on theoretical approach to follow) that are usually driven by “intuitive” introspection. According to Weger and Wagemann (2015, p. 45), the most important principles in neurophenomenology are (self-)reflection, observation, collection of detailed information before formulating hypotheses. Furthermore, researchers should practice extensively prior to participant recruitment, exchange experiences in introspective processes with colleagues and participants and–rather than generating only a single hypothesis–build multiple (opposing) hypotheses on causes and treatment of mental problems (Weger and Wagemann, 2015).

### Aim #4: (Neuro)Phenomenological Research: Assessment Instruments and Measurements for Public/Patient Involvement – Methods for Data Acquisition and Data Analysis

#### Data Acquisition Methods

Lifshitz et al. (2013, p. 1) note that researchers already use brain imaging and brain mapping techniques to a great extent, while methods for gathering first-person data are rarely employed. This section focuses on phenomenological methods to acquire experiential data that can be used to combine third person and first-person perspectives and presents requirements and constraints of these methods. Currently, a clear categorization of methods to acquire experiential data, and strong definitions and descriptions of their essential features do not exist.

The aim of this section is to provide an overview—besides of broader compilations on qualitative research (e.g., Flick, 2014)–of previously employed phenomenological methods that can be beneficial to investigate the subjective experience of a person with depression. Based on the reviewed studies, four methods for data acquisition and analysis are discussed: (1) experience sampling, (2) elicitation/explicitation interview, (3) photo elicitation, and (4) hypnosis.

Accessing experience is the main task in phenomenological research and several effective methods are available. Certain interview methods help accessing previously hidden experiences, such as using triggers in descriptive experience sampling or photo elicitation. Inducing a specific state of mind in the participants (as in hypnosis or meditation) can further help to foster meta-awareness and encourage accurate and detailed experiential reports. To study subjective experience of depression, suggested means of retrieving information are diaries, questionnaires or various interviews (Granek, 2006; Ratcliffe, 2015). Usually first-person descriptions are used, but other methods are also available. There are some phenomena that participants find difficult to discover on their own and, in such situations, they might need support from a “second person” who observes their behavior and non-verbal communication. This technique is commonly labeled as “elicitation/explication interview” (see Wertz, 2005, p. 171, for the example of denial of homophobia). Wertz (2005) proposes dialogues, interviews, group discussions, and simultaneous or retrospective descriptions of experiences in written or verbal form as additional potential methods to explore the subjective experience of depression. Still–regardless of the method used–to foster elaboration of experienced situations in daily life, and not opinions or inferences about a phenomenon, the focus must be set on the concreteness of the description provided by the person (Wertz, 2005, p. 171). The use of interviews is recommended for complex and subtle phenomena, and for participants who do not respond to simple question formats or test instructions (Wertz, 2005, p. 171).

### Experience Sampling

There are two prominent experience sampling methods. The descriptive experience sampling (DES), developed by Russell Hurlburt in the 1990s, describes inner experiences consisting of thoughts, feelings, visualizations, and their perceptual components (Hurlburt, 1997; Olivares et al., 2015). Participants are equipped with a beeper, an electronic device which emits a sound (Hurlburt and Heavey, 2004). The device is activated randomly (4–6 times per day) and prompts participants to focus their attention on the ongoing experience (Olivares et al., 2015). Participants externalize the experience verbally or through written and are further interviewed on it within 24 h. According to Hurlburt (1997, p. 68), the aim of DES is to spontaneously grasp and directly recognize, preconsciously emerging phenomena, inner thoughts, and feelings, or external images and sounds. This procedure distinguishes DES from the experience sampling method (ESM; Larson and Csikszentmihalyi, 1983) used in other fields of psychology (e g., Myin-Germeys et al., 2009). DES focuses on the qualitative aspect of experience without the attempt to quantify it via rating scales or structured questionnaires. Therefore, DES is considered a promising first-person method in studying depression (e.g., to discover, if rumination is a crucial aspect in intensifying and perpetuating depressive symptoms) thus supporting the development of preventative tools and treatment strategies (Scott, 2009; Olivares et al., 2015).

Telford et al. (2011) systematically examined the use of ESM in research on depression over a period of 25 years, and found that ESM, or comparable methodologies, contributes significantly to the understanding of this syndrome by fostering established theories, detecting new and clinically meaningful results, and formulating research questions. The authors, therefore, encourage the use of ESM for increasing knowledge on interpersonal pathoplasticity in the course of major depression (Telford et al., 2011).

### Elicitation/Explicitation Interview

Originally called explicitation interview, the elicitation interview technique was developed by Pierre Vermersch in the 1970s (Vermersch, 2009), but it was Claire Petitmengin who later described its methodological rules and showed its validity (Petitmengin, 2006). One of the core advantages in interviewing participants about their experiences is that a well-trained interviewer can support the process of finding expressions for events or sensations that the interviewed could otherwise hardly describe on his/her own (Petitmengin, 2006). Varela and Shear (1999, p. 10) describe the trained interviewer as an empathic resonator able to detect the way of thinking and reasoning of the client. Petitmengin (2006) suggests that specific techniques and principles, e.g., followed in the micro-phenomenological interview (Petitmengin et al., 2019) can ensure that the description obtained during the interview validly corresponds to the actual experience of the person. For example, the interviewer can stabilize the attention of the interviewed on the experience described or direct his/her focus toward different aspects or dimensions of the experience itself. The presenter involves the person to focus on the “how” rather than on the “what” and motivate the interviewed to provide detailed descriptions of a particular lived experience.

Whereas, the DES aims focus on random everyday experiences, the elicitation interview is interested in exploring specific experiences (Hurlburt, 2011). Further methodological differences between the elicitation interview and DES are discussed by Hurlburt (2011) and Olivares et al. (2015).

The search of the literature did not reveal the existence of studies, except a case-study (Depraz et al., 2017) using elicitation interview as method to access experiences in patients with depression, but evidence reveals that it is widely used in various fields ranging from education to performing arts (Maurel, 2009). It, therefore, represents a promising method to access a particular experience by focusing on procedural aspects. A study in the field of epilepsy showed that the experience-based anticipation of an epileptic seizure is possible and can be enhanced thereby consolidating the basis of cognitive treatment of epilepsy (Lutz, 2007; Petitmengin et al., 2007).

### Photo Elicitation

First mentioned by the photographer and researcher John Collier in 1957, photo elicitation is an interview method widely used in the fields of sociology and anthropology (Harper, 2002). A photograph or other forms of visual stimuli are employed during the interview as symbolic representations (Harper, 2002). Harper is interested in the evolutionarily older brain functions used in visual information processing rather than in verbal information processing. Therefore, images would evoke different conscious functions compared to words, as brain functions are used less extensively when exchanges of words alone take place than in the combination of processing images and words. Another important feature is that in-depth interviewing techniques require trust and understanding (Harper, 2002; Petitmengin, 2006). As argued by Harper (2002, p. 20), the interview situation is different when using photo elicitation, as both the researcher and the participant focus on an image. The gap between the participant's first-person experience and the researcher's observation is therefore significantly smaller than in classic interview situations. In the study by Sandhu et al. (2013), photo elicitation was used to explore the subjective experience of depression following a first psychotic episode. Participants were asked to take pictures representative of their feelings, which guided the creation of unstructured interviews aimed to explore emotions and feelings of the participants and to gain deeper insight on their experiences. The authors conclude that photo elicitation is a very helpful interview tool, as it “helped participants to visualize and verbalize their experiences” and to share histories authentically via articulation of abstract thoughts and feelings, anchoring these elements to specific life-memories of the participants (Sandhu et al., 2013, p. 172–173).

### Hypnosis

Lifshitz et al. (2013) argue for the use of hypnosis in phenomenological research, as it simultaneously meets the three phenomenological core essentials mentioned by Lutz and Thompson (2003, p. 5): facilitation of (1) altered states of awareness, (2) meta-awareness, and (3) experiential reports”. Lifshitz et al. (2013, p. 3) emphasize the enrichment of experiential processes by hypnotic and posthypnotic suggestions. The benefit of hypnosis–in comparison to meditation, another aspirant practice in neurophenomenological research (Lutz, 2007; Mackenzie et al., 2014; Ataria et al., 2015; Moss, 2015)–is that it does not require trained subjects. The elicitation interview described above is considered an enriching technique in the field of hypnosis, although phenomenologists do not recognize explicitation interviews as a form of hypnosis. Nevertheless, Lifshitz et al. (2013, p. 4) insist that the methods involve transformations of awareness using specific cultivated suggestions and propose explicitation interview as a thoughts-elicitating example of hypnosis-as-neurophenomenology (Lifshitz et al., 2013).

#### Data Analysis Methods

As long as there is a lack of well-established methodology in phenomenological research, it is crucial to find a way to interpret and connect to quantitative data. Lutz (2002, p. 1586) notes that phenomenology should develop adequate method of investigation of subjective experiences, and, to this aim Bockelman et al. (2013) recommend using concept mapping prior to experimentation, as well as glossary and framing, and conceptually sound summary to make sure that the analysis of the neurophenomenological data is well-prepared. Further more Petitmengin et al. (2019) recommended to also focus the role of interpretation in the analysis process, and follow certain specificities of e.g., micro-phenomenological analysis to guarantee the reproducibility of the analysis. Internal phenomenological consistency should be assured by iterative questioning processes and abstraction operations (Valenzuela-Moguillansky and Vásquez-Rosati, 2019).

### Narratives

In many studies (Smith, 1999; Granek, 2006; Rhodes and Smith, 2010; Sandhu et al., 2013; Ratcliffe, 2015), narratives are used to investigate contents and dynamics of conscious processes and gain a better understanding of affective disorders including depression (e.g., Ratcliffe, 2015).

Since “narrative” is a very broad term used in many different ways, Ratcliffe restricts it to “explicit autobiographical narratives of whatever length or sophistication, which relate life events in meaningful, chronologically structured ways” (Ratcliffe, 2015, p. 146). A narrative can be elicited and expressed in different forms and through different communication channels (verbal, written, etc.). Experience is reduced to a written text that is coded and interpreted by the researcher (Smith, 1999; Granek, 2006; Rhodes and Smith, 2010; Telford et al., 2011; Díaz, 2013; Sandhu et al., 2013; Fuchs, 2014), as well as prospectively combined with neuroscientific data.

Two interesting approaches to the analysis and categorization of narratives are introduced by Díaz (2013) with the aim to develop a method to study first-person data, or “phenomenological texts” as expression of conscious processes. Díaz (2013, p. 6) defines phenomenological texts as first-person verbalization of conscious states and experiences in the here and now and proposes the use of (1) computerized and rater-based interpretation systems to reliably detect “(a) perceptions/sensations, (b) affects, (c) thoughts (planning and recollecting) and (d) images (fantasies)” (Díaz, 2013, p8) in phenomenological reports. (2) Intersubjective analysis represents another method of interpreting experiential data that involves the agreement of different persons who function as judges; they first divide the text into segments, which can then be interpreted and assigned to different categories. Díaz (2013) used a text of the Spanish philosopher Unamuno (1970) and instructed sixteen students in psychology to structure the text into units, and to assign one or several of the above-mentioned categories (sensation, perception, affect, thought, image, recall, and intention) to each unit. These attributions are statistically processed to reveal significant intersubjective agreements. Díaz (2013, p. 10) assumes that intersubjective analysis is unique in allowing consensual definition of parts of a phenomenological text and examination of agreements on assigned mental categories. According to the author, although there are still important constraints (e.g., the gap between the conscious state of the writer and the text), psychic processes can be reliably rated in narratives” (Díaz, 2013, p. 11).

### Interpretative Phenomenological Analysis

Interpretative phenomenological analysis (IPA), a qualitative research method developed in the field of psychology by Jonathan Smith, explores how participants experience their personal world (Smith and Osborn, 2003; Smith, 2004; Larkin and Thompson, 2012). The common data collection method in IPA are semi-structured interviews, but personal accounts and diaries are also used. Advantages of semi-structured interviews are openness and flexibility as well as the establishment of empathy and rapport between the interviewer and the client (Smith and Osborn, 2003). According to Smith and Osborn (2003, p. 65), the level of transcription should be as close as possible to the actual conversation including all spoken words and false starts, pauses, laughs, etc. The text analysis proposed by Smith and Osborn (2003) can be divided into five stages: (1) repeated reading, (2) emergence of themes, (3) clustering of themes, (4) table of themes, and (5) writing up. First, the transcript is read several times during which interesting or significant statements are annotated. Smith and Osborn (2003) emphasize the importance of repeated reading in order to get familiar with the content, and also highlight the potential of new insights that each reading might provide. As in free textual analysis (close reading), strict operative rules are lacking in IPA, but Smith (2004) emphasizes that the interpretation must be clearly grounded in the text. There are also no regulations to assign meaning units to the text, but emerging themes must be chronologically ordered as they appear in the transcript. Connection can lead to new insights, so that some themes can be clustered together, while others appear as subordinate concepts. The more themes emerge the richer certain text passages are. This is an iterative process, during which the researcher continuously compares the connections made within different themes to the actual words of the respondent. Once clusters are identified, they are ordered and listed with their subordinated in a table of themes. If more than one participant is interviewed, the first analysis can either be used to orient subsequent analysis or work as scratch. Finally, the table of content is used as a basis to put the insights extracted from the participants accounts into a narrative argument. Here, it is important to clearly distinguish between verbatim extracts from the transcript and its interpretation.

In contrast to nomothetic approach, which predominates in mainstream psychology and allows to make probabilistic assumptions, the idiographic method characteristic of IPA allows specific assertions about the individuals to be made. Furthermore, IPA is inductive–meaning that it is flexible enough to allow unanticipated themes to emerge–and interrogative, as it is combining quantitative data and therefore questions existing results (Smith, 2004).

### Discussion

To our knowledge, this paper presents the first comprehensive and structured summary of theoretical and empirical research on first-person approaches to the study of the mind and depression. It starts by discussing theoretical assumptions and considerations supporting the inclusion of experiential dimension in the study of the mind, while arguments and criticisms to materialistic and reductionistic approaches, e.g., neuroscience also emerged.

For the examination of cognitive and affective processes and phenomena, the exclusion of the first-person perspective seems especially detrimental. Some studies emphasize the importance not to omit first-person experiences from the classification of psychiatric disorders and criticize reductionistic tendencies. When it comes to psychopathology, the inclusion of the subjective experience becomes essential, and the use of phenomenological approaches appears especially fruitful. A phenomenological approach supports a more reliable and patient-centered diagnosis, thus more effective treatment. While the phenomenological approach is criticized because it can alter experience and lead to confabulation, researchers employing phenomenological methods stress that these methodological drawbacks can overcome or be reduced by training participants with methods that support the elicitation of (past) experiences.

In the second part of this review, frameworks that combine first- and third-person accounts, are discussed, and references for choosing adequate study design, participants, and researchers based on their skills and qualifications are presented. According to the literature, neurophenomenological studies with trained participants, or experimental studies that include data on first-person experiences are advised. The selection of participants depends on the study design, the research aim, and the specific method employed. Depending on the general framework, it is recommended either to train participants in observing and describing their subjective experiences (some researchers recommend participants being experienced in psychoanalysis), or to train researchers who are open to interdisciplinary research in supporting phenomenological reflections of participants.

Eligible methods to acquire experiential data are also discussed, including experience sampling, elicitation or explicitation interview, photo elicitation, and hypnosis. These phenomenological techniques are widely applied in empirical studies and can be helpful for the reporting of subjective experience in patients with depression. While the experience sampling method supports participants in observing and reporting their experiences on their own (photo), elicitation interviews and hypnosis make use of a second person who supports individuals in eliciting and describing first-person experiences. For the analysis of experiential data, it is recommended to convert the acquired data into narratives. Narratives are presented as a product of data acquisition and interpretative phenomenological analysis is discussed as a favorable data analysis method.

As there are no explicit frameworks, nor specific guidelines for the conduction of neurophenomenological studies in the field of mental health, this review covers theoretical as well as methodological aspects discussed in previous contributions on the application of phenomenological approaches within the clinical context. This might have led to the exclusion of other potentially suitable phenomenological approaches, but that have not yet been applied or discussed in the field of metal health. Empirical research that incorporates first- and third-person accounts to the study of mental disorders are scarce, a systematic review is of course lacking. Building upon theoretical assumptions and considerations, this review provides an overview of available methods that can complement third person research and provides references and recommendations on how to include the first-person perspective in a research study. Future systematic research on the use of holistic approaches and participatory research designs for the study of cognitive and affective phenomena of psychiatric illness are encouraged.

## Data Availability Statement

The original contributions presented in the study are included in the article/Supplementary Material, further inquiries can be directed to the corresponding author/s.

## Author Contributions

KB and IP designed the study, conducted extensive literature searches, analyzed the data, and wrote the first draft of the paper. HL-S revised and wrote the manuscript. GP and TP reviewed methodological as well clinical issues and further edited the manuscript. All authors approved the final version of the manuscript.

## Conflict of Interest

The authors declare that the research was conducted in the absence of any commercial or financial relationships that could be construed as a potential conflict of interest.

## Footnotes

^1^In this contribution the term (first-person) experience agrees with the definition by Parnas and Zahavi (2002, p. 145), who describe it as a conscious state that is combined with subjective feelings and meanings.
