# Supplementary material for: How to Include Patients' Perspectives in the Study of the Mind: A Review of Studies on Depression
Source: Front Psychol. 2021 Apr 12;12:651423. doi: 10.3389/fpsyg.2021.651423 (PMC8072288; doi:10.3389/fpsyg.2021.651423)
Supplement: Supplementary file 1 [file Table_1.docx]

**Supplementary table 1**: Search strategies

| **Database** | **Key terms used for search** | **Number of results** | **Number of selected articles** |
| --- | --- | --- | --- |
| PubMed | “neurophenomenology” AND “method” (all fields), all years | 17 | 9 |
|  | “depression” AND “neurophenomenology” | 6 | 1 |
|  | “MDD” AND “phenomenology” | 34 | 5 |
|  | “phenomenology” AND “depression” AND “subjective experience” | 14 | 3 |
| Taylor & Francis Online | “+neurophenomenology +method” (all fields), Journals | 64 | 26 |
|  | “phenomenology” AND “depression” in Abstract, Journals | 72 | 13 |
| Wiley Online Library | “neurophenomenology” AND “method”  (all fields), Journals | 83 | 7 |
|  | “phenomenology” AND “depression” in Abstract, Journals | 87 | 22 |
| SAGE journals | “neurophenomenology” AND “method”  (all fields) | 57 | 9 |
|  | “phenomenology” AND “depression” in Abstract | 0 | 0 |
| ScienceDirect | “neurophenomenology” AND “method”  (all fields) | 68 | 17 (19) |
|  | “phenomenology” in Abstract AND “depression” in Abstract, Journals | 177 | 36 |
| BioMed Central | “neurophenomenology AND method”  (all fields) | 3 | 2 |
| JSTOR | “neurophenomenology AND method”  (all fields) | 12 | 0 (1) |
|  | “phenomenology AND depression” in Abstract | 1 | 1 |
| **SUM** | | 695 (100%) | 151 (21,73%) |

*Note*. The number in brackets in the column “Number of selected articles” indicates the total number of selected articles including overlapping (i.e., articles also found in another database) results.
